# Gastroenterology Training and Career in Women: Challenges and Opportunities

**DOI:** 10.1002/ueg2.12764

**Published:** 2025-02-14

**Authors:** Eleni Manthopoulou, Ignacio Catalan‐Serra, Maura Corsetti, Andrea Nowak, María Jesús Perugorria, Cem Simsek, Carolina Ciacci

**Affiliations:** ^1^ Digestive and Interventional Endoscopy Unit ASST Grande Ospedale Metropolitano Niguarda Milan Italy; ^2^ Gastroenterology Department of Medicine Levanger Hospital Nord‐Trøndelag Hospital Trust Levanger Norway; ^3^ Department of Clinical and Molecular Medicine Norwegian University of Science and Technology Trondheim Norway; ^4^ Centre of Molecular Inflammation Research NTNU—Norwegian University of Science and Technology Trondheim Norway; ^5^ NIHR Nottingham BRC Nottingham University Hospitals NHS Trust and the University of Nottingham Nottingham UK; ^6^ Nottingham Digestive Diseases Centre Translational Medical Sciences School of Medicine University of Nottingham Nottingham UK; ^7^ United European Gastroenterology Headquarters Governance & Community Management Vienna Austria; ^8^ Department of Liver and Gastrointestinal Diseases Biogipuzkoa Health Research Institute Donostia University Hospital University of the Basque Country (UPV‐EHU) Donostia‐San Sebastian Spain; ^9^ CIBERehd Instituto de Salud Carlos III (ISCIII) Madrid Spain; ^10^ Department of Medicine Faculty of Medicine and Nursing University of the Basque Country UPV/EHU Donostia‐San Sebastián Spain; ^11^ Department of Medicine Division of Gastroenterology Hacettepe University Faculty of Medicine Ankara Turkey; ^12^ Department of Medicine Surgery and Dentistry Scuola Medica Salernitana University of Salerno Baronissi Salerno Italy

**Keywords:** authorship, career, endoscopy, gender, grants, impostor syndrome, training

## Abstract

This review highlights gender gaps in training, career, and work‐life balance in gastroenterology. Stereotypes and biases toward women’s abilities and commitment to their careers can influence evaluations, advancement in gastroenterology training, and career progression. The findings indicate that lack of or limited access to mentorship and sponsorship, as well as support networks, can hinder the professional development of women. Moreover, results indicate that we must improve work‐life balance measures, for example offering flexible working hours and compensation and support for women during pregnancy, after childbirth, and motherhood. However, reports on such equity measures are scarce, and we lack scientific evidence of their impact. This review concludes that to reduce gender gaps and make a positive impact, we need educational and promotional programs and monitoring of their outcomes.

## 1 Introduction

The present narrative review of the limited sources available explores gender disparities within GI training, careers, and work‐life balance and examines measures to compensate for them.

## 2 Methods

The authors conducted an extensive literature search utilizing the PubMed, Web of Science, and Embase databases, focusing on articles published in English between 2010 and June 2024.

**FIGURE 1:**
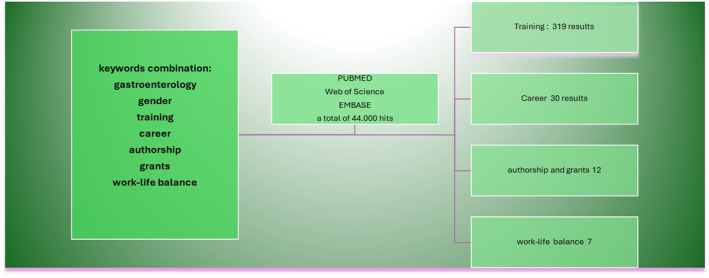
Flowchart of findings of the literature search.

The selected search terms were *gastroenterology* and *gastrointestinal diseases*, in conjunction with *gender, training, authorship, career,* *grants*, and *impostor syndrome*; only original papers in English were included. Figure 1 shows the literature research flowchart.

The literature search revealed three primary areas of interest: GI training, careers (hospital and academic), and work‐life balance. These fields serve as focal points for examining potential gender disparities and evaluating the existing evidence base within gastroenterology.

## 3 Results

### 3.1 The Uphill Struggle of Training in Gastroenterology

Women will encounter considerable obstacles such as gender bias, family planning concerns, inflexible schedules, radiation exposure, and ergonomically inadequate equipment [1, 2]. The combination of these factors and insufficient representation and mentorship leads to an intensified struggle and, therefore, the discouragement of women in the field [2]. According to Rembacken et al., female trainees were primarily concerned about childbearing and motherhood. Despite the requirement for full‐time employment, they discovered that many institutions failed to provide appropriate job‐sharing alternatives. For example, some countries did not offer protection for training assignments during maternity leave. Many women felt fatigued throughout lengthy endoscopic operations and were unable to handle specific equipment [3]. Similarly, South Asian and Indian female trainees experienced a higher rate of career interruptions, primarily due to pregnancy and childcare provision [4].

Sensible pregnancy accommodation may include fewer overnight calls and endoscopic work, adequate colleague coverage, and financial support. Despite advancements in paternity leave, women continue to shoulder more childcare responsibilities due to societal standards, economic constraints, and personal preferences [1]. Parental leave policies should be available to all genders. On top of that, revised return‐to‐work fellowship schedules, childcare assistance, and anti‐bias programmes can help reduce postpartum professional challenges [5].

Correspondingly, various surveys conducted in Europe, the Americas, and Asia identified part‐time work during training as an important option for female trainees [1, 2, 3, 4, 6, 7]. For example, the British Society of Gastroenterology education study in 2020 reported 29.5% of women working flexible hours, compared to 2.6% of men [1], while Akbani et al. indicated that overall employment flexibility could bring greater benefit, as it also improves diversity among GI staff [6]. Multiple studies imply that part‐time work, flexible hours, reduced commutes, finding job options closer to residence, and taking family leave are practical solutions for promoting women in training [7]. Limiting trainees’ work hours while allowing them to extend their training or move to a competency‐based fellowship [4] and eliminating the requirement for additional training during leave periods to acquire essential skills can improve work‐life balance for everyone [1].

Apart from the challenge of integrating family life, sadly, tangible gender discrimination persists in gastroenterology training, with female trainees frequently receiving unequal treatment [3]. Formal mentorship is a good complementary solution and can significantly affect success, as a woman‐orientated gastroenterology mentorship plan likely enhances personal achievement, resilience, and contentment, and reduces burnout, according to Smith et al. [8]. Male and female mentors alike should be promoted and supported to feel more comfortable mentoring opposite‐gender physicians. Interestingly, Rabinowitz LG found that women were more likely to prefer a female mentor [9]. As many women share the challenging experience of maintaining their personal lives while combating gender bias and unequal treatment in their institutions, same‐sex mentoring may provide an added benefit for them [9].

Moreover, endoscopy‐related injuries pose an increasing and substantial challenge for all endoscopists today. For instance, Pawa et al. indicated that the incidence of injuries is evenly distributed between the two genders [10]. In contrast, in a United States survey published in 2024, the female gender has been identified as an independent risk factor for injury [11]. Most gastroenterologists reported endoscopy‐related injuries, with women having a higher risk (63.4%) than men (40,9%) [11].

Furthermore, only 16.7% of women were satisfied with the endoscopic equipment’s ergonomics, compared to 55.7% of men [12]. Female participants reported a higher incidence of upper extremity injuries, whereas male participants reported more occurrences of low back discomfort [13]. On average, women in the study possessed smaller hand sizes and shorter stature [13]. These physical traits, together with the reduced skeletal muscle mass in female participants and the availability of only one‐size endoscopes, suggest that female subjects are likely to employ different approaches in endoscopy compared with male subjects [13]. Endoscopists of all genders and abilities benefit from ergonomic equipment and proper training that reduces injuries. However, endoscopy instructors must be educated to teach ergonomic methods while considering trainees’ physical stature and hand size [9]. Consulting an authority in the field, the European Society of Gastrointestinal Endoscopy (ESGE), in their recently published DEI position statement, clearly recommends the implementation of ergonomic principles and adapted workplace conditions for all personnel [14].

### 3.2 Career in Gastroenterology: A Laborious Achievement

#### 3.2.1 Academic Career

Successful academic gastroenterology careers encompass awards and grants, research and authorship, and leadership positions.

According to the UNESCO Institute for Statistics, women account for only 30% of the world’s researchers. Therefore, in 2024, UNESCO launched a campaign to reduce gender gaps in research and academic careers, including GI [15]. The observed gender academic gap is attributed not only to a lack of female mentors’ differential participation in grant applications and success rates [16] but also to the fact that only about 30% of first authors in scientific manuscripts are women [17, 18].

The American Association of Medical Colleges reports that female gastroenterologists have nearly doubled in 12 years. However, despite this growth, they still struggle to attain leadership in fellowship programmes and academic roles [19]. An evaluation of award recipients from three major United States gastroenterology and hepatology scientific societies revealed that only 8.4% were given to women [20]. Meanwhile, a 2009 UK survey found that female first authors of scientific publications increased from 10.5% in 1970 to 36.5% in 2004, but female senior authors only increased from 12.3% to 16.5% [21].

The under‐representation of women among researchers [15] may cause or be the effect of gender inequality in grants. A systematic review conducted between 2005 and 2020, encompassing 55 studies mainly from Europe and North America, revealed that although women applied for and accepted fewer grants, the acceptance rates were comparable across genders, indicating an absence of bias in peer‐reviewed outcomes [16]. Nevertheless, women received smaller and fewer awards after reapplying. Studies conducted outside the United States demonstrated a 6% greater award rate than studies conducted within the United States.

Other reviews also identified limitations of the current literature, as it lacks consistency in defining sex and gender as well as data by year, suggesting a need for systematic reviews based on funder reports [16, 22]. They highlight that it is insightful and beneficial when funders publish success rates by sex, race, and ethnicity, as displayed in the National Institutes of Health Data Book via NIH RePORTER [23].

Whether the difference between US‐based and non‐US‐based studies reflects that many European countries have gender equality laws and policies in place is unclear. Europe has promoted gender equality in research funding, such as Horizon Europe [24] and the Marie Sklodowska‐Curie Actions, reporting 53.2% of projects awarded to women from 2014 to 2018 [25]. In contrast, the World Economic Forum reported in 2020 that the United States ranked 53^rd^ in gender equality among 153 countries [26]. Regarding grant review panels, a recent study conducted at the United States Veterans Health Administration (VHA) Health Services Research and Development explored the relationship between reviewers and awardees regarding female gender and racial and ethnic minority individuals. This cross‐sectional study found that although women were well‐represented on most review panels, racial and ethnic minority individuals were underrepresented. These were more likely to be awarded by review panels with higher proportions of women and racial and ethnic minority reviewers [27].

Research often neglects the role of social identities in shaping discrimination and disadvantage [22]. PROGRESS‐PLUS of the Cochrane Collaboration identifies characteristics such as gender, race, and socioeconomic status that stratify opportunities and outcomes [28]. In evaluating women’s roles in research, one should also consider other valuable yet often unnoticed metrics, such as “personal being”, mentorship, emotional support, and sponsorship, which are predominantly contributed by women [29].

Regarding the lack of women in leadership positions, one can look at scientific societies, for example, UEG, which may mirror the overall phenomenon. During 32 years of existence, the UEG had 22 presidents, only one of them a woman (term 2022–2023, elected position/vice‐president 2020–2021). The current secretary general (term 2022–2025) and the secretary general elected for 2026–2029 are women [30]. The trend of women applying and being elected for leadership positions gained momentum 4 years after the inauguration of the E&D Group in 2015, which provides sponsorship for women aspiring to become leaders (UEG internal data).

### 3.3 Hospital Career

To illustrate, a survey of 564 out of 1220 invited Italian gastroenterologists revealed that only 4.6% of female respondents held a leading position, compared to 15,4% of male respondents. Determining factors included lack of mentorship, less competitive behavior of women, and work‐family reconciliation for young female doctors. Interestingly, only 18% believed that proactive inclusion measures were indicated to close the gender gap [31].

Surveys reported that one‐third of female physicians faced disadvantages in attending advanced or interventional endoscopy masters and expressed feelings of gender‐biased underappreciation [32]. Women gastroenterologists perceived disadvantages compared to men in career opportunities and salary negotiations. A study showed that 32% of men earned a salary over $600,000 compared to 3% of women, with 68% believing their salary did not match their qualifications. Female gastroenterologists felt less respected and were perceived as more demanding than their male colleagues [33]. Similarly, a survey conducted in India and other South Asian countries revealed remarkable gender disparities in training and career advancement. Among respondents, 40.7% of female gastroenterologists felt gender bias negatively impacted their careers compared with 1.5% of males, and 44.7% of women reported a salary gap existed versus 29.1% of men [4].

Given these disparities, female gastroenterologists may be prone to being unsatisfied with their work and suffering psychological disorders. The Impostor Syndrome in high‐achieving professional women refers to persistent self‐doubt and fear of being exposed as a fraud, leading individuals to attribute success to external factors like luck rather than skill, thus reinforcing feelings of unworthiness and anxiety about being exposed as incompetent. Fahra et al. reported burnout rates of 64.4% in women and 45.8% in men, with impostor syndrome being prevalent among female gastroenterologists [34]. To cope with self‐doubt and inadequacy, female gastroenterologists may engage in problematic habits such as perfectionism, the quest for ultimate expertise, self‐reliance, false beliefs in innate brilliance, and the myth of the “superwoman” [35].

Coping with gender‐biased discrimination and managing internal obstacles such as impostor syndrome adds up to a considerable mental load that requires unseen and unrewarded labor invested by many women forging their careers in gastroenterology.

### 3.4 Work‐Life Balance: The Need to Crack the Mystery

Over time, the discourse on work‐life balance has produced more differentiated expressions, such as work‐life integration, work‐family reconciliation, and career‐family equilibrium, to better reflect the often challenging but indeed continuous task of adapting and integrating one’s professional, personal, and social life. To crack the mystery, one must acknowledge that only part of this delicate balance is upheld by individual exertion and that systemic, equitable measures are essential to perform the feat in a sustainable way.

Fortunately, finally, work‐life balance is increasingly recognized as a critical factor in physician well‐being, productivity, and career satisfaction. Although binary gender roles have evolved, we see that when it comes to parenthood and care work, in heterosexual partnerships, the traditional role division overall persists, as women still take on more care work [1].

Studies across medical specialties have consistently shown lower work‐life balance satisfaction among women physicians than men in terms of making sacrifices in either of life’s domains still seems inevitable. A recent United States survey indicated that 67% of women in GI felt that the maternity leave they were granted was insufficient for recovery and bonding. The most common reasons for taking a shorter leave were financial concerns, followed by fears of delayed graduation, negative impacts on promotion, pressure from administration or seniors, personal choice, and concerns about regression of technical skill [36].

Furthermore, the gender disparity in work‐life balance may be attributed to combined factors, namely a lack of flexibility and support in the workplace, as well as discrimination and unconscious biases [37]. Examples of unconscious, stereotype‐based gender bias are expectancy bias, for example, presuming the lack of women is a pipeline issue, prescriptive gender norms, e.g. believing women do not have requisite leadership skills, and female self‐confidence. Such biases can perpetuate inequities in pay and promotion and create an unsupportive work environment. Conversely, gender‐bias‐habit‐changing interventions can result in measurable positive change [38].

### 3.5 Proposed Interventions to Reduce Gender Inequities in Gastroenterology

Laver et al. [39] systematically reviewed interventions supporting women’s academic careers, emphasizing the importance of cross‐disciplinary networks, unbiased grant funding, and equity metrics. The review emphasizes that “bottom‐up” strategies, such as mentorship, are useful but difficult to evaluate. Academic institutions should, therefore, adopt “top‐down” interventions, like the UK Athena Scientific Women’s Academic Network (SWAN) Charter [40]. Another example is the University of Texas HEAL + Plus program for under‐represented groups, significantly boosting residents’ confidence in securing academic positions. In 2019, only 3% of 32 residents felt confident about obtaining an academic role, but 67% of program participants later secured academic faculty positions [41]. The selection of candidates only by portfolio and/or interview may lead to biases and prejudices. A centrally organized entrance exam, coupled with an interview, could be more equitable and exempt from subjectivity for both genders, minorities, and underrepresented people. Grant funding agencies should also be obliged to eliminate biases in the application process, and scientific societies must promote and report progress in equity and diversity.

**FIGURE 2:**
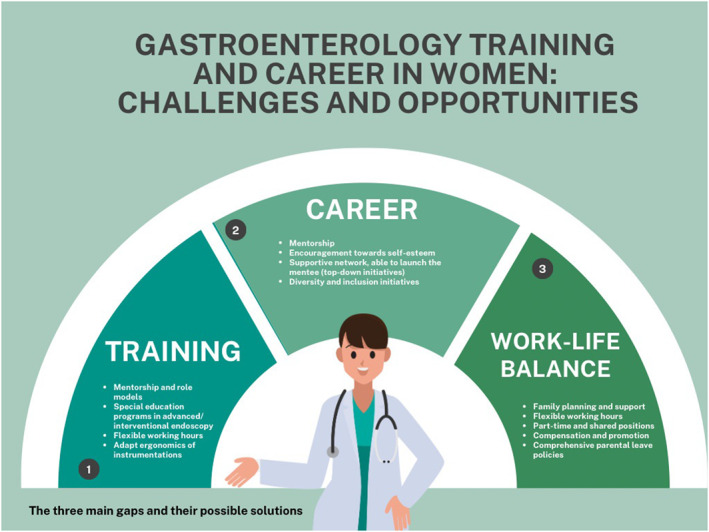
Central proposed interventions to fill the gender gap in gastroenterology.

Flexible work arrangements, parental leave for all genders, diversity initiatives, anti‐bias training, and equitable promotion practices are key to achieving gender balance. Figure 2 shows the three key focus areas of gender inequalities—Training, Career, and Work‐Life Balance—and summarizes potential solutions for each.

Further research and institutional accountability are crucial for improving women’s recruitment, retention, and advancement in gastroenterology.

## 4 Conclusion

Gastroenterology poses distinct challenges for women, marked by gender disparities in training, career advancement, and work‐life balance. Solving these issues requires top‐down interventions. Gender equity can be further enhanced by early career development and anti‐bias training facilitated by multi‐institutional networks. Current evidence is limited, primarily from studies conducted in the United States, with insufficient attention to non‐binary gastroenterologists. Thorough research is crucial for examining gender disparities and assessing interventions. Establishing a supportive and inclusive environment in the gastroenterology community fosters all physicians’ well‐being and professional success.

## Conflicts of Interest

The authors declare no conflicts of interest.

## Data Availability

Data sharing is not applicable to this article as no new data were created or analyzed in this study.
